# Genetic evidence of population subdivision among Masai giraffes separated by the Gregory Rift Valley in Tanzania

**DOI:** 10.1002/ece3.10160

**Published:** 2023-06-12

**Authors:** George G. Lohay, Derek E. Lee, Lan Wu‐Cavener, David L. Pearce, Xiaoyi Hou, Monica L. Bond, Douglas R. Cavener

**Affiliations:** ^1^ Biology Department Penn State University University Park Pennsylvania USA; ^2^ Research Innovation for the Serengeti Ecosystem, Grumeti Fund Mara Tanzania; ^3^ Wild Nature Institute Concord New Hampshire USA; ^4^ Department of Ecosystem Science and Management Penn State University University Park Pennsylvania USA; ^5^ Department of Evolutionary Biology and Environmental Studies University of Zurich Zurich Switzerland

**Keywords:** conservation, evolutionary significant units, genetic connectivity, genomics/proteomics, Gregory Rift, Masai giraffe, wildlife corridors

## Abstract

The Masai giraffe has experienced a population decline from 70,000 to 35,000 in the past three decades and was declared an endangered subspecies by the IUCN in 2019. The remaining number of Masai giraffe are geographically separated by the steep cliffs of the Gregory Rift escarpments (GRE) in Tanzania and Kenya dividing them into two populations, one west and one east of the GRE. The cliffs of the GRE are formidable barriers to east–west dispersal and gene flow and the few remaining natural corridors through the GRE are occupied by human settlements. To assess the impact of the GRE on Masai giraffe gene flow, we examined whole genome sequences of nuclear and mitochondrial DNA (mtDNA) variation in populations located east (Tarangire ecosystem) and west (Serengeti ecosystem) of the GRE in northern Tanzania. Evidence from mtDNA variation, which measures female‐mediated gene flow, suggests that females have not migrated across the GRE between populations in the Serengeti and Tarangire ecosystems in the past ~289,000 years. The analysis of nuclear DNA variation compared to mtDNA DNA variation suggests that male‐mediated gene flow across the GRE has occurred more recently but stopped a few thousand years ago. Our findings show that Masai giraffes are split into two populations and fulfill the criteria for designation as distinct evolutionary significant units (ESUs), which we denote as western Masai giraffe and eastern Masai giraffe. While establishing giraffe dispersal corridors across the GRE is impractical, conservation efforts should be focused on maintaining connectivity among populations within each of these two populations. The importance of these efforts is heightened by our finding that the inbreeding coefficients are high in some of these Masai giraffe populations, which could result in inbreeding depression in the small and fragmented populations.

## Graphical abstract

The steep escarpments of the GRV are formidable barriers to migration of Masai giraffe. To assess the impact of the GRV on Masai giraffe gene flow, we examined nuclear and mitochondrial DNA variation in subpopulations east and west of the GRV in Tanzania. Strikingly, evidence from mtDNA variation suggests that females have not migrated across the GRV between major subpopulations in the Serengeti and Tarangire ecosystems since the approximate time that Masai giraffes emerged as a (sub)species 250 kya. However, male‐mediated gene flow across the GVR has occurred over most of the history of Masai giraffes but may have ceased in the recent past as natural migratory routes across the escarpment have been blocked by human activities. These findings suggest that the Masai giraffe is now split into two separate metapopulations and in the absence of gene flow will eventually become separate species with populations of <20,000.

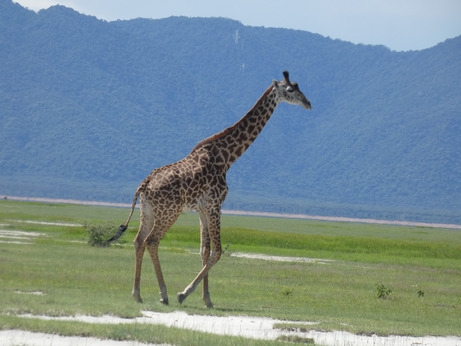

## 1 INTRODUCTION

As a result of human activities wild mammal populations have declined over the past 10,000 years and now account for <4% of mammal biomass on the planet, with humans, pets, and livestock constituting ~96% (Bar‐On et al., 2018; Ritchie et al., 2022). During the past few decades, the charismatic megaherbivores on the African continent, including giraffes (*Giraffa camelopardalis*), elephants (*Loxodonta cyclotis* and *L. africana*), and rhinoceroses (*Diceros bicornis* and *Ceratotherium simum*), have experienced dramatic reductions in population sizes, population fragmentation, and the threat of extinction (IUCN, 2022). The causes of the massive decline in mammals—especially megaherbivores—are numerous and complex but all appear to stem from human activities including conversion of natural habitats to agriculture and human settlements, diverting and depleting water sources, legal and illegal hunting, and human‐induced climate change (Ripple et al., 2015).

Recent population genomic analysis of the major giraffe subspecies suggested that the decline in giraffe population abundance began soon after the separation of distinct subspecies and their dispersal across sub‐Saharan Africa during the middle Pleistocene (Coimbra et al., 2021) in parallel with a rapid decline of all ruminants (Chen et al., 2019) and an increase in human populations, hunting, and introduction of zoonotic diseases from livestock. The viral disease rinderpest was introduced to the African continent in the 1890s and caused several mass mortality events for ruminant wildlife including giraffes over a period of 70 years (Plowright, 1982). More recently, the global giraffe population declined 36%–40% (from 1985 to 2015) as a consequence of human activities, with <100,000 individuals remaining (Muller et al., 2018). The Masai giraffe (*G. c. tippelskirchi*), found in southern Kenya and throughout Tanzania, declined by 50% in three decades to approximately 35,000 individuals and was listed as an endangered subspecies in 2019 (Bolger et al., 2019).

**FIGURE 1:**
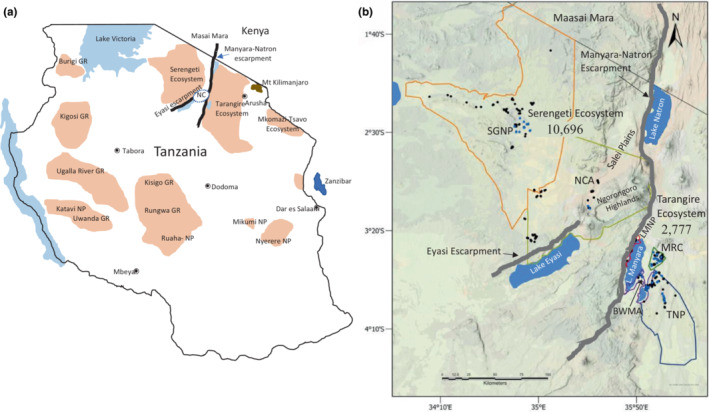
Masai giraffe (*Giraffa camelopardalis tippelskirchi*) distribution in Tanzania and study areas in the Serengeti and Tarangire ecosystems. (a) Distribution of major populations in Tanzania (shaded) with names of national parks (NP) and game reserves (GR) and ecosystems (boundaries not shown). Masai giraffe populations extend north from the Serengeti ecosystem to the Masai Mara in Kenya and from the Mkomazi GR into the Tsavo NP in Kenya. The location of the Manyara‐Natron escarpment and the Eyasi escarpment of the Gregory Rift system bisect Masai giraffe populations in the Serengeti and Tarangire. (b) Study area including the Serengeti National Park (SGNP) and Ngorongoro Conservation Area (NCA) west of the Manyara‐Natron and Eyasi escarpments and the Tarangire National Park (TNP), Manyara Ranch Conservancy (MRC), Burunge Wildlife Management Area (BWMA), and Lake Manyara National Park (LMNP) east of the escarpments. The Ngorongoro Highlands together with the Manyara National Park and Eyasi escarpment pose a formidable barrier to terrestrial wildlife movements. Populations census numbers are shown for the Serengeti ecosystem (10,696) and the Tarangire ecosystem (2777) from Bolger et al. (2019).

Geographically, the steep cliffs of the Gregory Rift Escarpments (GRE; including the Manyara‐Natron, and Eyasi Escarpments) bisect the Masai giraffe populations in northern Tanzania into two distinct regions: west of the GRE including the Serengeti Ecosystem and east of the GRE including the Tarangire Ecosystem (Figure 1a). In total <14,000 Masai giraffe are reported to exist in these two ecosystems (Figure 1b) (Bolger et al., 2019). The Serengeti Ecosystem (~33,000 km^2^) and the Tarangire Ecosystem (~25,000 km^2^) are two of the most critical ecosystems in Tanzania for biodiversity conservation. Both ecosystems conserve biodiversity and large landscapes, and support two of Africa's few remaining long‐distance migrations of large mammals including the white‐bearded wildebeests (*Connochaetes taurinus*) and the plains zebras (*Equus quagga*) and along with their major predators including lions (*Panthera leo*) and leopards (*Panthera pardus*) (Bond et al., 2022; Estes, 2014; Guy et al., 1981; Hopcraft et al., 2013; Lamprey, 1964; Lohay et al., 2022; Morrison et al., 2016; Prins & de Jong, 2022; Sinclair, 2012). Each ecosystem also host two of the largest remaining populations of Masai giraffes (Figure 1b), (Bolger et al., 2019; Lee & Bolger, 2017; Lee & Bond, 2022; Lee & Strauss, 2016; Strauss et al., 2015), with substantial contributions to vegetation dynamics, food webs, and community ecology.

In regions without geographic barriers Masai giraffes can roam over large areas, with mean home range of 114 km^2^ for females and 157 km^2^ for males (Knüsel et al., 2019), including among‐population movements across unprotected human‐altered lands in the protected areas of the Tarangire Ecosystem east of the GRE (Bond, Lee, et al., 2021; Lee & Bolger, 2017). By contrast Masai giraffe populations in the Serengeti Ecosystem west of the GRE are dispersed over a large single, protected landscape mostly free of human settlements and agriculture, although whether giraffes move between dispersed populations in the Serengeti Ecosystem is unknown. However, overall wildlife movements within Tanzania have been constricted by rapid expansion of agriculture and human settlements (Caro & Davenport, 2016; Jones et al., 2009; Lamprey, 1964; Riggio et al., 2022; Riggio & Caro, 2017). Although savannah elephants are known to cross the GRE (Douglas‐Hamilton, 1973; Lohay et al., 2020; Prins et al., 1994), most other wildlife movements east–west or around the formidable cliffs of the GRE are rare (Baker et al., 1972; Scoon, 2018). Because the escarpments of the Gregory Rift were well established by 1 mya (Macgregor, 2015) and prior to the emergence of the Masai giraffe as distinct species, the presence of Masai giraffes on the western and eastern sides of the GRE strongly suggests that at some point in their evolutionary history Masai giraffes migrated across or around the escarpments or that they were possibly founded independently from another source population.

A critical question germane to the long‐term conservation and management of Masai giraffes is whether the populations located east and west of the GRE are now reproductively isolated and unable to maintain genetic diversity across the populations through dispersal and gene flow. Further, populations within the eastern and western regions are growing more fragmented and may be losing the capacity for genetic exchange (Lee et al., 2023). Population genetic analysis provides a means to ascertain gene flow between populations, and female‐ vs. male‐mediated gene flow can be assessed by comparing mitochondrial DNA (mtDNA) variation vs. nuclear DNA variation because mtDNA is strictly maternally inherited whereas nuclear DNA is inherited from both parents (Allendorf, 2017; Allendorf et al., 2010). An earlier study of the population genetics of major giraffe subspecies (Brown et al., 2007) based on a small fragment of the mtDNA and a small number of microsatellite nuclear markers showed significant differentiation among populations in southern Kenya and northern Tanzania across the Gregory Rift Valley and suggested that the Masai giraffe may constitute more than one species. The objectives of our study were to determine if the steep escarpments impacted genetic differentiation between Masai giraffe populations that occur east and west of the GRE, and to measure genetic exchange among populations, and levels of inbreeding within populations, in each region (Figure 1b). To address these questions, we employed a suite of analytic tools to conduct a population genetic analysis of whole genome sequencing (WGS) data of the mtDNA, and nuclear genomes of population samples obtained from both sides of the GRE in the Serengeti Ecosystem and the Tarangire Ecosystem in northern Tanzania and assessed potential giraffe dispersal routes across the GRE based on maximal slopes.

## 2 MATERIALS AND METHODS

### 2.1 Study sites

Our study sites included the Serengeti and Tarangire ecosystems located in northern Tanzania (Figure 1b). Surveys that were conducted between 2011 and 2015 estimated 10,696 and 2777 Masai giraffe in the Serengeti and Tarangire‐Manyara regions, respectively (Bolger et al., 2019). Our study area within the Tarangire Ecosystem consisted of four protected areas including Tarangire National Park (TNP), Manyara Ranch Conservancy (MRC), Lake Manyara National Park (LMNP), and Burunge Wildlife Management Area (BWMA), which is immediately contiguous with the TNP (Figure 1b). Manyara Ranch Conservancy (MRC) is a unique wildlife area in Tanzania, as it does not fall in the categories of formal protected areas. Rather, MRC is an open area supported by the African Wildlife Foundation for wildlife conservation and livestock keeping, and functions as part of a wildlife corridor between the TNP and the LMNP and the Lake Natron area (Bond et al., 2022). BWMA, located between the TNP and the LMNP, is a community‐based conservation initiative started about 20 years ago by several villages (Lee, 2018). The BWMA is used for promoting eco‐tourism and provides habitat for several wildlife species. The BWMA is also part of the corridor connecting the TNP and the MRC and Lake Natron (Kiffner et al., 2020; Lee, 2018). The BMWA giraffe samples for whole genome mtDNA and nuDNA were included with the Tarangire National Park samples because they are geographically adjacent, Masai giraffe freely move between the TNP and BWMA, and no geographic obstacles impede movement. The Serengeti National Park (SGNP) and the Ngorongoro Conservation Area (NCA) form a major part of the Serengeti Ecosystem. While the SGNP is reserved for photo tourism and wildlife management, the NCA allows tourism and pastoralism. Over the past few years, the number of livestock and humans has increased within the NCA (Catherine et al., 2015).

### 2.2 Fecal sample collection

We obtained fecal samples from 320 Masai giraffe in six protected areas in Tanzania including the TNP, BWMA, MRC, LMNP, the Malanja depression of the NCA, and the Seronera area of SGNP (Figure 1b) between December 2019 and March 2021. Within each protected area, we sampled giraffes from several localities typically separated by more than 1 km to reduce the probability of sampling highly related individuals. Once giraffes were sighted, we observed and waited for them to defecate. We photographed each giraffe, recorded its sex, and estimated its age using morphological characteristics after (Strauss et al., 2015). We collected giraffe fecal samples as soon as possible after defecation because giraffe pellets dry quickly. We collected the epithelial cells adhering to the outside layer of pellets (2–4 pellets). We used a razor blade to scrape/peel the thin outer layer from each pellet and placed it into a 50 mL tube. We added Queen's College buffer (Ahlering et al., 2012) immediately into the tube containing samples.

### 2.3 Tissue sample collection

We used remote biopsy darts to obtain tissue samples from 100 giraffes from five protected areas including the TNP, BWMA, MRC, the Malanja depression of the NCA, and the Seronera area of SGNP. We recorded the GPS location where we collected each sample. Darting was performed by a trained veterinarian from the Tanzania Wildlife Research Institute. We used the remote biopsy device Pneu‐Darts type U (3cc) (Pneu‐Dart, Inc.), shot from a Model 196 Pneu‐Dart cartridge‐fired projector, to obtain skin biopsies. The type U Pneu‐darts are designed to collect a 3cc tissue plug and then immediately drop off the animal. The veterinarian aimed the rifle at flat surfaces on thighs or shoulders from a distance between 10 and 30 m. We removed the tissue samples from the needles, placed them in a 2 mL microcentrifuge tube, and secured the tube in a cool box with ice packs and froze them to −20°C within 6 h of collection. The samples were collected from different giraffe groups within a short time to minimize sampling closely related individuals and by choosing animals with dissimilar coat spot patterns and/or separated by a 1‐km distance. Animals with similar coat patterns are more likely to be related (Lee et al., 2018). To ensure that we have unique individuals, we matched photographs of spot patterns from the biopsy samples using WILDID software to detect replicates (Bolger et al., 2012).

### 2.4 DNA extraction, PCR amplification and sanger sequencing of mitochondrial DNA fragments

We extracted fecal DNA using the QIAamp PowerFecal DNA kit (QIAGEN) and isolated tissue DNA with the Monarch Nucleic Acid Purification Kits using the manufacturer's protocol, but we increased incubation time to 12 h. to ensure the whole tissue was completely lysed. We extracted DNA from the samples at the Nelson Mandela African Institution of Science and Technology.

We PCR amplified 1140 nt segment of cytochrome b gene (Bock et al., 2014). We performed PCR amplification using at least 10 ng of DNA template, 0.5 μL of 10 μM of both primers, 7.5 μL of 2× GoTaq master mix (Promega), and 3 μL of DNA template. We performed the PCR reaction with the initial polymerase activation step at 95°C for 3 min, denaturation at 95°C for 30 s, annealing temperature at 58°C for 45 s, and extension at 72°C for 30 s for 35 cycles. We sequenced the PCR products by Sanger sequencing using both forward and reverse primers at the Pennsylvania State University Huck Institute's genomic core. We visually inspected sequence results in the trace file format using SnapGene® software 4.2.4 (from GSL Biotech). Clean sequences were aligned with a previously published sequence of a giraffe from Tanzania and Kenya (Agaba et al., 2016; Bock et al., 2014; Brown et al., 2007; Coimbra et al., 2021). We trimmed sequences and collapsed haplotypes using FaBox (Villesen, 2007). We calculated haplotype diversity (H_d_) and nucleotide diversity (π) using DnaSP whereas pairwise genetic fixation (*F*_*ST*_) was calculated using the Arlequin version 3.5 (Excoffier & Lischer, 2010). We constructed a median‐joining network using PopArt 4.8.4 (Leigh & Bryant, 2015). To evaluate the relationship between population pairwise *F*_*ST*_ and geographic distance, we used two methods: regression of *F*_*ST*_/1−*F*_*ST*_ on geographic distance (km) (Rousset, 1997b) and the Isolation by Distance (IBD) Mantel test (Bohonak, 2002; Jombart, 2008), which is based on the correlation between Slatkin's linearized pairwise *F*_*ST*_ and geographical distance (Slatkin, 1993).

### 2.5 Whole genome sequencing

To assess nuclear genetic variation, we analyzed the whole genome sequence (WGS) of the 100 Masai giraffe dart biopsy samples using short read Illumina sequencing. We sequenced 25 samples at a medium coverage level (~15–25× total reads) and 75 samples at low coverage level (2.5× total reads). Low coverage genomic sequence data are sufficient to accurately estimate most population genetic parameters utilizing genotype likelihood estimations as described below. To assess mitochondrial genetic variation, we extracted and assembled the whole mitochondrial genome (16,430 nucleotides) for the 100 dart biopsy samples. Uniquely indexed libraries for each of the samples were prepared at the Pennsylvania State University Huck Institute's genomic core and sequenced at the Pennsylvania State University Hershey genomics core on an Illumina NovaSeq. The samples were prepared with unique index sequence attached to the template. All samples were sequenced at the Sequencing facility in Hershey Medical Center, as 150 bp paired‐end reads on a multiplexed sequencing NovaSeq Illumina instrument. Bcl2fastq software was used to demultiplex the mixed sequences. During the demultiplexing, reads were checked against the adapter sequence (‐‐mask‐short‐adapter‐reads = 10) and the bases run into the adapter were turned to Ns. The 75 samples sequenced at low coverage yielded ~1.66× average peak depth of mapped reads and the 25 samples sequenced at medium coverage yielded ~24.1× average peak depth of mapped reads. In order to avoid the magnitude difference in coverage in the population analyses, we down‐sampled the 25 medium coverage reads by 85% using the random sample process (seed = 11) in seqkit v0.11.0 (Shen et al., 2016). The final average coverage of these 25 down‐sampled samples was ~3.09× average peak depth of mapped reads.

### 2.6 Raw DNA sequence processing and quality control

We evaluated the raw DNA genomic sequences using FastQC v0.11.8 (Andrews, 2010; Li & Durbin, 2009). The mean quality score for all sample sequences ranged between 34.78 ~ 35.34. FastQC Per Base Sequence Quality showed all bases with the exception of the last base of each read had a quality score higher than 24. Only three samples whose 90th percentile quality score at the last base were above 10, while other samples' were above 18. We kept all bases in the analyses. Since any read whose end overlapped the adaptor had been masked by bcl2fastq, the FastQC Adapter Content report indicated “no adapter found”. Fastp v0.20.0 (Chen et al., 2018) was used to check overrepresented sequences and to confirm that it was unnecessary to perform the adapter removal step.

### 2.7 Reference genome

We used the Masai giraffe genome assembly ASM165123v1 (GCA_001651235.1) that was previously reported (Agaba et al., 2016), and improved by using HiC data to generate chromosomal level assemblies (Dudchenko, 2019) employing the Jucier assembly methods (Dudchenko et al., 2017; Durand et al., 2016). We used the sequences from the fourteen autosomes and sex chromosome from the HiC assembly (ASM165123v1_HiC.fasta.gz) as the reference genome and mapped DNA sequence reads of each individual giraffe to the chromosome assemblies.

### 2.8 Preparation of alignment files

We aligned the paired‐end reads against the reference using the BWA‐MEM algorithm of BWA v0.7.17‐r1188 (Li & Durbin, 2009) with the default values for mapping scoring/screening and algorithm settings. The option of ‐M was used to mark the shorter split hits as secondary for downstream duplicate marking. The output SAM files of 75 low coverage samples were sorted by coordinates and converted to bam format using Samtools v1.15.1 (Li et al., 2009) followed by marking the duplicate alignments using Picard MarkDuplicates (Broad Institute, 2019) in GATK4.2.2.0 (van der Auwera et al., 2013). For the 25 samples that were down‐sampled, the alignment outputs were first filtered of unmapped reads and then sorted by sequence name into bam format with Samtools. Subsequently, we removed duplicates using the Picard MarkDuplicates tool with both “REMOVE_DUPLICATES” and “REMOVE_SEQUENCING_DUPLICATES” flags set to true. The resulting bam files were then sorted again by coordinates. We generated the final genome alignment and coverage statistics using Samtools stats and BEDTools (Quinlan & Hall, 2010).

### 2.9 Inbreeding coefficient

We estimated individual inbreeding coefficients *F* using ngsF (Vieira et al., 2013). The individual inbreeding coefficient here is defined as the proportion of the sites across the genome of that individual, where the observed alleles are identical by descent. The ngsF program requires a binary genome likelihood input file in BEAGLE format. We generated genotype likelihood (GL) files using ANGSD v0.939‐10 command from input bam files with “‐doGlf 3” as the output option. These bam files were filtered to exclude reads that failed vendor quality checks, that were nonuniquely mapped, whose mate was not mapped, and whose mapping quality was below 30. The mapping quality in indel regions was adjusted with the flag “‐C 50” to adjust mapping quality containing excessive mismatches. All the bases with base quality below 30 were discarded. The aforementioned processes of filtering were applied to the bam files, which were then used as input to calculate GL in the entire study. We estimated GL using GATK (McKenna et al., 2010) model (‐GL 2). The GATK model applies Bayesian statistics to estimate the most likely genotype based on joining the base probability from all reads that cover the target base. The base probability was associated with the base quality of the reads in the bam files. The major and minor alleles were inferred from GL (doMajorMinor 1). Only the biallelic sites were used to calculate per site frequencies (‐skipTriallelic 1). The sites with MAF below 0.05, or with a *p*‐value larger than 1e−6 were also excluded. The output GL files were uncompressed for running ngsF. The ngsF consists of a two‐step workflow: an approximated EM algorithm (‐‐approx_EM) and a full implementation of EM ML algorithm. The former produces an estimate of all parameters such as the individual inbreeding and site frequency, which is required as the initial values to start the iterations in the latter. To avoid the algorithm converging to local maxima, the approximated EM was run 20 times with a convergent criteria (‐‐min_epsilon) of 1e−5 and random generated initial values (‐‐init_values r) in each run. We chose the parameters file of the run that exhibited the largest global log likelihood as the initial values in the full EM ML run (without ‐‐approx_EM flag) where a convergent criterion of 1e−9 was used.

### 2.10 Population structure

We conducted principal component analyses on all samples using PCAngsd (Meisner & Albrechtsen, 2018). This program applies a novel approach of estimating individual allele frequencies to compute a covariance matrix. It relaxes the assumption of a conditional independence between individuals given the population allele frequency. PCAngsd requires a GL input file in a BEAGLE genotype likelihood format. We calculated the GL using the same steps as described in the inbreeding coefficient calculations except replacing the output flag of “‐doGlf 3” with “‐doGlf2”. The covariance matrix file (.cov file) was converted into eigenvectors and plotted using the R version 4.2 (R Core Team, 2021). We estimated the individual admixture proportions with NGSadmix v32 (Skotte et al., 2013). Two parameters were implemented to determine the convergence: log likelihood difference in 50 iterations (‐tolLike50) and tolerance for convergence (‐tol). Default values of 0.1 and 1e−5, respectively, were used in this calculation. The same input GL files for PCAngsd were used in this analysis. We computed admixture for *K* from 1 to 5 for five subpopulations where *K* is the number of ancestry states. The log likelihood of each *K* was extracted from the output log file and plotted against the *K* value. From the plot, the value of *K* was determined as the point at which the slope of line segments shifts downward. The result from NGSadmix (.qopt file) contains the inferred proportions for each individual and was plotted using the R package “xadmix” (Schönmann, 2022).

### 2.11 Population genetic differentiation

To estimate genetic differentiation between the populations from different regions, we calculated the *F*_*ST*_ using ANGSD (Korneliussen et al., 2014) for each population pair. The calculations consist of two steps for each population. First, we estimated the sample allele frequency (SAF) likelihood using ANGSD (‐doSaf 1). Then, the results from the first step were used to estimate the folded (with ‐fold 1 flag) site frequency spectrum (SFS) using realSFS function in the ANGSD package. We calculated the *F*_*ST*_ of each population pair using both sample allele frequency likelihoods of the populations and their pairwise SFS as priors in the “fst index” subprogram. The *F*_*ST*_ was calculated using the “fst stats” subprogram of realSFS.

### 2.12 Phylogenetic analyses of mtDNA haplotypes

We used Clustal Omega (Sievers & Higgins, 2021) to generate a nexus alignment of whole genome mtDNAs of representatives of each of the major giraffe subspecies and major mtDNA haplogroups of Masai giraffe. We performed phylogenetic analysis and tree construction using the IQ‐tree stochastic algorithm and maximum likelihood method (Trifinopoulos et al., 2016) and 5000 bootstrap replicates. The results were plotted using the ETE tree viewer (Huerta‐Cepas et al., 2016). We estimated divergence time of mitochondrial haplotypes by Bayesian analysis of molecular sequences related by an evolutionary tree (BEAST, version 2.7.3), which uses the Markov chain Monte Carlo method (MCMC) to average over tree space (Bouckaert et al., 2019). To calibrate the molecular clock to the time of the most recent common ancestor (TMRCA), we used the TMRCA estimate of 11.5 MYA for *Giraffidae* (divergence of giraffe and okapi, *Okapia johnstoni*) (Agaba et al., 2016) and 1 MYA for the *Giraffa* subspecies (Petzold & Hassanin, 2020). The program Tracer was used to evaluate the Bayesian/MCMC estimates, and the data were plotted using Figtree v1.4.4 (Rambout, 2018).

Brown and coworkers (Brown et al., 2007) had previously investigated mtDNA variation in the major giraffe subspecies including Masai giraffes sampled from several locations in Kenya and Tanzania using a small segment (654 bp) of the mtDNA genome. In addition, mtDNA whole genome sequence had been determined for five Masai giraffe in the Selous Game Reserve in Southern Tanzania (Coimbra et al., 2021) and one Masai giraffe from Maasai Mara in Kenya (Agaba et al., 2016). We compared our results and determined haplotype equivalencies among these studies by focusing on a 652 nt fragment that had been sequenced in all studies.

### 2.13 Dispersal route assessment

To ascertain the degree to which the steep escarpments of Gregory Rift may impede giraffe dispersal between the Serengeti and Tarangire ecosystems, we performed a slope assessment of the Manyara‐Natron escarpment along its 400+ km length from south central Kenya to north central Tanzania and the Eyasi escarpment that bifurcates from the Manyara‐Natron escarpment in the Ngorongoro highlands and terminates ~100 km near the southwest end of Lake Eyasi (Figure 1b). To evaluate the slopes across the escarpments, we generated elevation profiles at 5 km intervals along each escarpment and determined the maximal slope for a perpendicular transect over a 1–5 km distance across the escarpment. We estimated maximal slopes using Google Earth Engine (Gorelick et al., 2017), which provides accurate estimates of slope comparable to GIS but with more efficient deployment (Safanelli et al., 2020; Yu et al., 2021). For the Manyara‐Natron escarpment, the 0 km elevation profile transect (EPT) was located in Kenya at 1°15′08.11″S 35°59′41.71″E and the 400 km terminal EPT was located in Tanzania at 4°42′54.32″S 35°56′59.97″E. The 0 km and 100 km EPT for the Eyasi escarpments were at 3°19′43.14″S 35°16′46.22″E and at 3°47′55.20″S 34°30′27.33″E, respectively. In addition, high resolution satellite images were visually examined along the entire lengths of both escarpments to identify local regions where maximum slopes dropped below 40% and/or the presence of animal tracks crossed over the escarpment; we denoted these sites as potential wildlife escarpment passes (WEP) to distinguish them from wildlife corridors through human imposed barriers. We determined pathways through these potential WEPs, as well as the Kitete‐Selela wildlife corridor (3°14′02.55″S 35°54′57.35″E) (Jones et al., 2009) by mapping pathways to minimize slope across the escarpment. If animal tracks were seen, we mapped minimal slope pathways along these tracks.

Genetic distance and geographic distance between populations with restricted dispersal are predicted to be positively correlated giving rise to isolation by distance (Slatkin, 1993; Wright, 1943). To determine if the Masai giraffe populations exhibited significant isolation by distance, we performed pairwise correlation analyses of genetic and geographic distance for three alternative dispersal routes identified as minimal slope passes across the Manyara‐Natron escarpment of the Gregory Rift. We mapped these alternative dispersal routes to minimize slopes along their entirety to provide the least resistance to animal movement. Specifically, using satellite view in Google Earth, we drew tracks using the elevation slope function to avoid geographic obstacles (e.g., mountains, hills, ravines, lakes, rivers, and streams). Among the authors (GL, DL, MB, and DC) at least one of us have also driven through all of the areas included in the tracks. We denoted these tracks as least resistance paths. In addition, we assessed the default Euclidean strait line transects between each population.

## 3 RESULTS

### 3.1 Population genetic analysis of mitochondrial genome sequence

**FIGURE 2:**
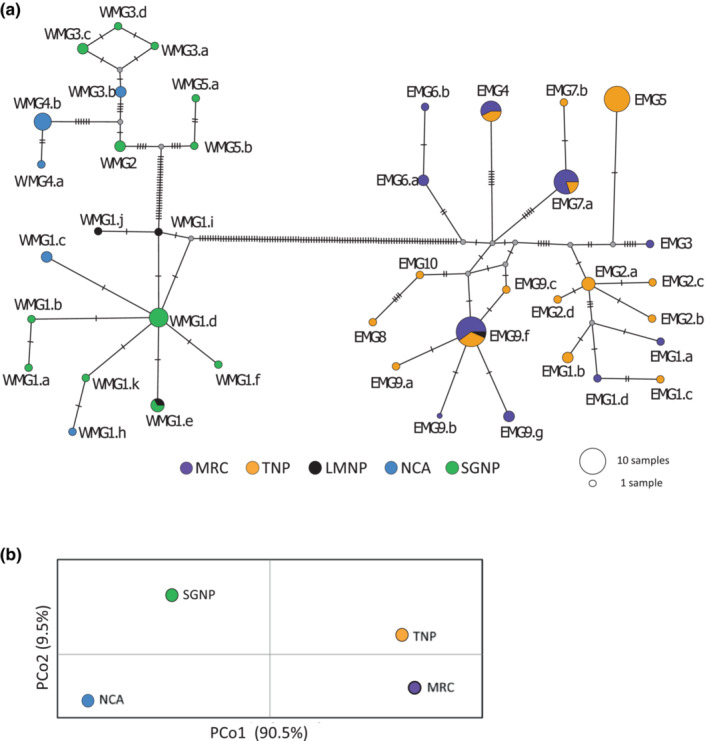
Mitochondrial whole genome sequence analysis of Masai giraffe haplotypes. (a) Neighbor joining network showing genetic differentiation of the Masai giraffe mtDNA haplotypes. Haplotypes present in western and eastern Masai giraffe are denoted as WMG and EMG, respectively. The size of circles corresponds to haplotype frequencies and hatch marks represent the number of mutations/nucleotide differences between haplotypes. Colors represent population location and fraction of each haplotype. (b) Principal coordinate analysis (PCoA) of mtDNA population differentiation.

**FIGURE 3:**
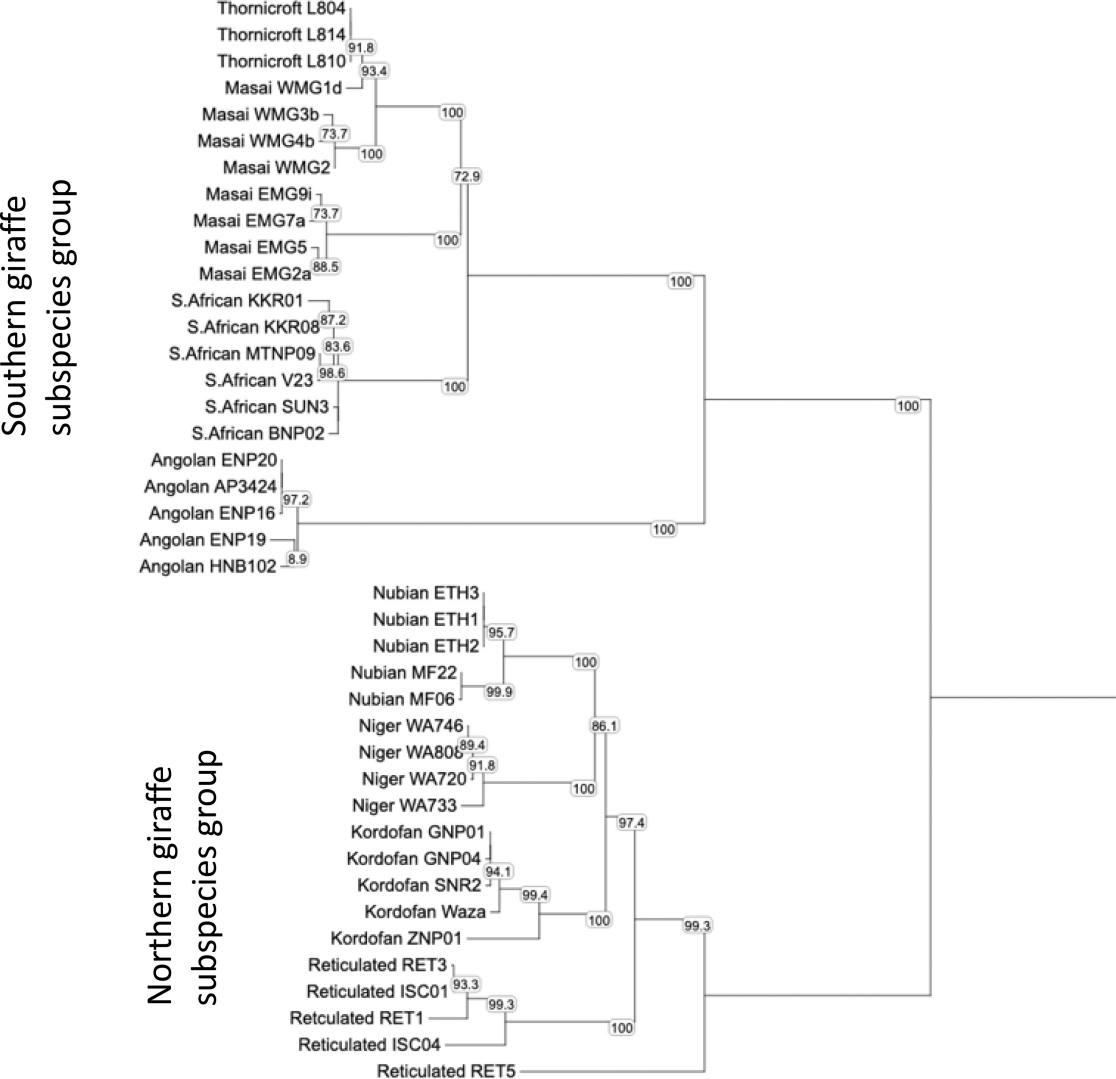
Mitochondrial whole genome trees of the major giraffe subspecies including Masai (*G. c. tippelskirchi)*, Thornicroft's (*G. c. thornicrofti*), South African (*G. g. giraffa*), Angolan (*G. g. angolensis*), Reticulated (*G. c. reticulata*), Kordofan (*G. c. antiquorum*), Niger (*G. c. peralta*), and Nubian (*G. c. camelopardalis*). Numbers indicate bootstrap support for each branch node, with values >95 considered as statistically significant. The bootstrap value for the branch node between Masai and South African giraffe is only 72.3% and, therefore, not statistically significant.

To assess mtDNA variation we sequenced a 1140 bp fragment for 320 individuals (Table S1) and the entire 16,430 bp mtDNA genome for 100 individuals collected from six Masai giraffe populations in the Serengeti and Tarangire ecosystems (Figure 1b). From the 100 mtDNA whole genome sequence (mtWGS) samples we identified 54 unique haplotypes among the six populations (Figure 2a), which were inclusive of all the 13 unique haplotypes found among the 320 individuals sequenced for only the 1140 nt mtDNA fragment (Table S2). Among the 100 whole genome mtDNAs sequence, we identified 14 haplotype clades with subclade members of a group differing by no more than 3 bp from each other. The 14 mtDNA haplotype clades exhibit an extreme geographic sorting with 13 of the groups found exclusively west or east of the GRE (Figure 2a). A dominant mtDNA clade (WMG1) in the Serengeti Ecosystem populations was also found in several individuals in LMNP but was not found in any other Tarangire Ecosystem population. We denoted these two distinct mtDNA clades as Western Masai Giraffe (WMG) and Eastern Masai Giraffe (EMG). The WMG and EMG mtDNA clades differed by 100 nt or more (Figure 2a) revealing a remarkable degree of genetic differentiation. To ascertain the relative age and origin of these two major haplogroups, we compared them to the mtDNA whole genome sequence of other giraffe subspecies. We found that the WMG and EMG haplogroups are nearly as similar to South African giraffe (*G. c. giraffa*) haplotypes as they are to each other (Figure 3). The statistical analysis of the tree topology cannot distinguish between two sequential bifurcation events (Masai and South African haplotypes separating followed by the emergence of the Masai WMG and EMG) and a single trifurcation event giving rise to all three simultaneously. We estimated the EMG‐WMG haplotype divergence to the most common recent ancestor using two alternative calibrations. The estimated EMG‐WMG time to the most recent common ancestor (TMRCA) was 665 kya using *Giraffidae* (divergence of giraffe and okapi most recent common ancestor) as the calibration time and 289 kya using *Giraffa* (i.e., divergence of the southern and northern giraffe subspecies most recent common ancestor).

We also found that all seven of the Thornicroft's giraffes (*G. c. thornicrofti*), which mtDNA whole genome sequence data are available, exhibited the WMG1 haplotype (Figure 3). Moreover, the Masai giraffe WMG haplotypes are more closely related to the Thornicroft haplotypes than they are to eastern Masai giraffe haplotypes. The WMG1 haplotype clade found in 70% of the WMG samples and Thornicroft's giraffe appears to be the ancestral western Masai giraffe haplogroup with the other four WMG clades showing substantial divergence from it (Figure 3). The closely related Thornicroft's mtDNA haplotypes and the WMG1.d haplotype also showed the lowest genetic distance for all pairwise comparisons with all other southern and north giraffe subspecies clades (Table S3). WMG1 was also the lone haplogroup found among individuals in the LMNP population (Figure 2a). Interestingly, three different WMG1 haplotypes found in LMNP differed 1–3 nt from each other, and two of them were unique and not found in western populations (Figure 2a). The nine eastern Masai giraffe haplotype clades exhibited a more star‐like phylogenetic relationship with nearly equal genetic distances between them.

To quantify mtDNA population differentiation we estimated *F*_*ST*_ for mtDNA haplotypes for all pairwise Masai giraffe populations in this study (Table 1). *F*_*ST*_ for mtDNA showed relatively low values between populations on the same side of the Gregory Rift escarpments compared to pairwise comparisons of populations across the GRE, as expected given the very large differences between the western and eastern Masai giraffe haplotype clades. The principal coordinate analysis (PCoA) also revealed a large degree of differentiation on the PCoA1 axis between populations lying east and west of the GRE (Figure 2b).

**TABLE 1:** Pairwise *F*_*ST*_ estimates for whole genome mtDNA (below the diagonal) and for whole genome nuDNA (above the diagonal).

	MRC	TNP	NCA	SGNP
MRC	—	0.0140	0.0832	0.0795
TNP	0.0037	—	0.0775	0.0733
NCA	0.3692	0.3569	—	0.0491
SGNP	0.2429	0.1853	0.0686	—

MRC = Manyara Ranch Conservancy, TNP = Tarangire NP, NCA = Ngorongoro Conservation Area, SGNP = Serengeti NP.

**FIGURE 4:**
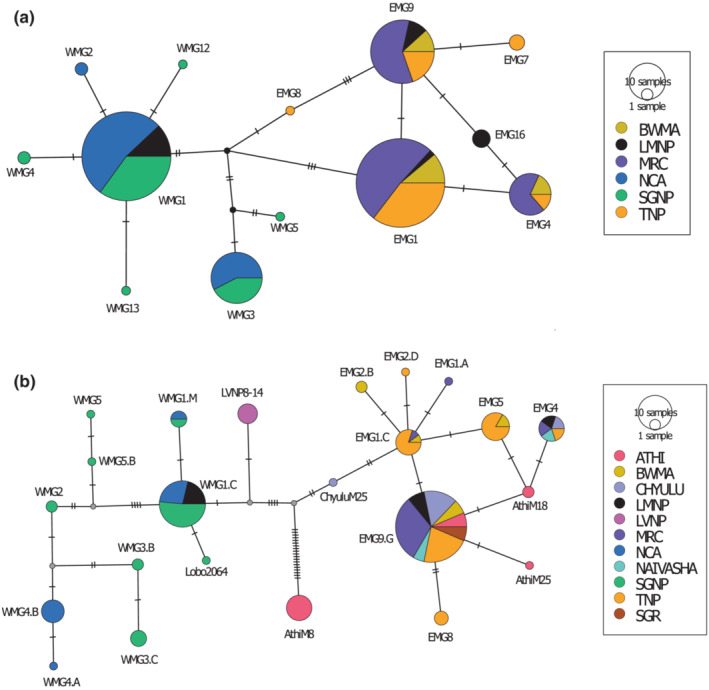
Mitochondrial DNA haplotype neighbor joining network for (a) 1140 bp fragment of 320 Masai giraffe samples. (b) 652 bp mtDNA haplotypes for 194 Masai giraffes. Giraffes from Athi, Chyulu and Naivasha in Kenya (Brown et al., 2007) carry haplotypes that are clustered in the EMG subclade.

Brown and coworkers (Brown et al., 2007) had previously investigated mtDNA variation in the major giraffe subspecies including Masai giraffe sampled from several location in Kenya and Tanzania using a small segment (654 bp) of the mtDNA genome. In addition, mtDNA whole genome sequence had been determined for five Masai giraffe in the Selous Game Reserve in Southern Tanzania (Coimbra et al., 2021) and one Masai giraffe from Maasai Mara in Kenya (Agaba et al., 2016). To compare our results and determine haplotype equivalencies among these studies, we focused on a 652 nt fragment that had been sequenced in all studies. The overall haplotype network showed the same large separation of western and eastern Masai giraffe haplotype clades (Figure 4; Table S4). Five additional unique haplotypes, one in the Serengeti (Lobo) and four in East Rift populations in southern Kenya were found in the samples from Brown et al. (2007). Four of these unique haplotypes differed by only 1 or 2 nt from other haplotypes, but one haplotype, found only in the Athi River Ranch, exhibited 22 nt difference from next most similar haplotypes (Figure 4b). The Athi River Ranch mtDNA haplotype was previously reported to be closely related to a reticulated giraffe (*G. c. reticulata*) haplotype, which may have resulted from an introgression event (Petzold & Hassanin, 2020). We also compared the whole genome mtDNA sequence of our 100 samples with whole genome mtDNA sequences obtained from Masai giraffes in the Selous Game Reserve (Coimbra et al., 2021) and Maasai Mara (Agaba et al., 2016) used in the small mtDNA fragment analysis described above. These giraffes exhibit whole genome mtDNA haplotypes that are identical or nearly so to one of the 14 mtDNA haplotype clades described herein. As predicted from their location relative to the Gregory Rift escarpments, the Maasai Mara giraffe exhibited a western Masai giraffe haplotype whereas the Selous Game Reserve giraffes exhibited eastern Masai giraffe haplotypes (Figure 4).

### 3.2 Nuclear DNA variation

**FIGURE 5:**
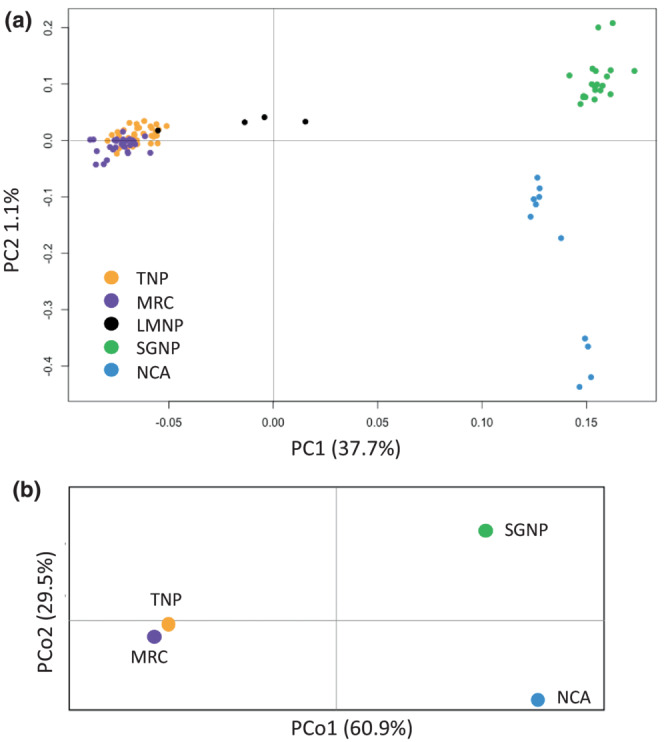
Genetic differentiation of whole genome nuDNA SNPs. (a) Principal component analysis (PCA) and (b) Principal coordinate analysis (PCoA).

To examine population differentiation of nuDNA variation, we estimated *F*_*ST*_ for all pairwise populations in our study (Table 1) excluding LMNP because its sample size (4 animals) was too small. *F*_*ST*_ values were estimated using sites shared between each population pair, which ranged from 1.31–1.91 million single‐nucleotide polymorphism (SNP) sites. The Tarangire NP (TNP) and Manyara Ranch (MRC) giraffes exhibited the smallest *F*_*ST*_ (0.0140) whereas the highest *F*_*ST*_ values (0.0773–0.0832) were between the four western and eastern Masai giraffe pairwise populations that lie across the Manyara‐Natron escarpment from each other (Table 1). Principal component analysis (PCA) showed that individuals from the western and eastern populations formed two distinct clusters with no intermixing (Figure 5a). The four LMNP giraffe showed intermediate PC1 values between EMG and WMG. Principal coordinate analysis (PCoA) exhibited the same large separation of western and eastern Masai giraffe populations on coordinate 1 axis and large separation of SGNP and NCA on coordinate 2 axis (Figure 5b).

**FIGURE 6:**
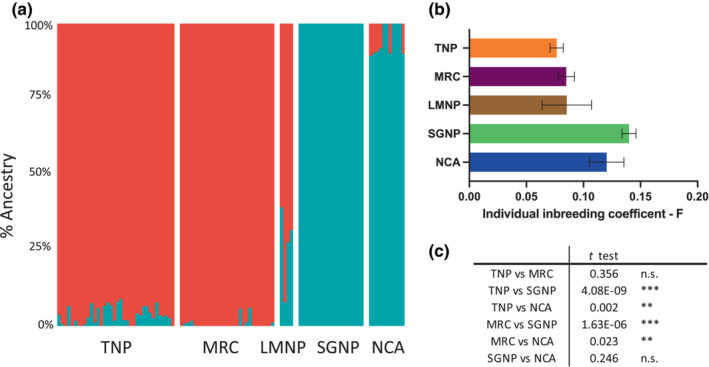
Population structure and inbreeding estimates of 100 individual Masai giraffe located among five populations (a) Structure/Admixture analysis each bar represents the ancestry percent of two clusters (red and blue). (b) Individual inbreeding coefficient averaged for each of the five populations. (c) Students *t*‐test for significant differences in mean inbreeding coefficient between populations. See Table S6 for inbreeding coefficients for each individual. **p<0.02, ***p<0.001.

Population structure and admixture analysis showed the presence of two distinct clusters with *K* = 2 the best fit to the data (Figure S1). All the SGNP and NCA giraffes were clustered together and all the MRC and TNP giraffes clustered together with at least 90% ancestry (Figure 6a). <10% admixture (fraction of shared membership with the opposite cluster) was seen among these individuals. Although the four LMNP individuals clustered most closely with the EMG populations, three of them showed substantial admixture (25%–40%) from WMG.

### 3.3 Inbreeding coefficient

We estimated individual inbreeding coefficients, *F* (Vieira et al., 2013) for each of the 100 WGS nuDNA samples, which are based on a total of 2,054,254 SNPs across the 14 autosomes (Figure 6b, Table S6). Individual inbreeding coefficients ranged between 0 and 0.218 and the average was 0.078 across all samples and populations. The average inbreeding coefficient for each population revealed significant differences between eastern and western Masai giraffe populations with WMG populations exhibiting approximately 1.6‐fold higher *F* than EMG populations (Figure 6b). No significant differences in the average *F* were seen within EMG or WMG populations.

### 3.4 Dispersal routes and geographic connectivity of the Tarangire and Serengeti ecosystems

**FIGURE 7:**
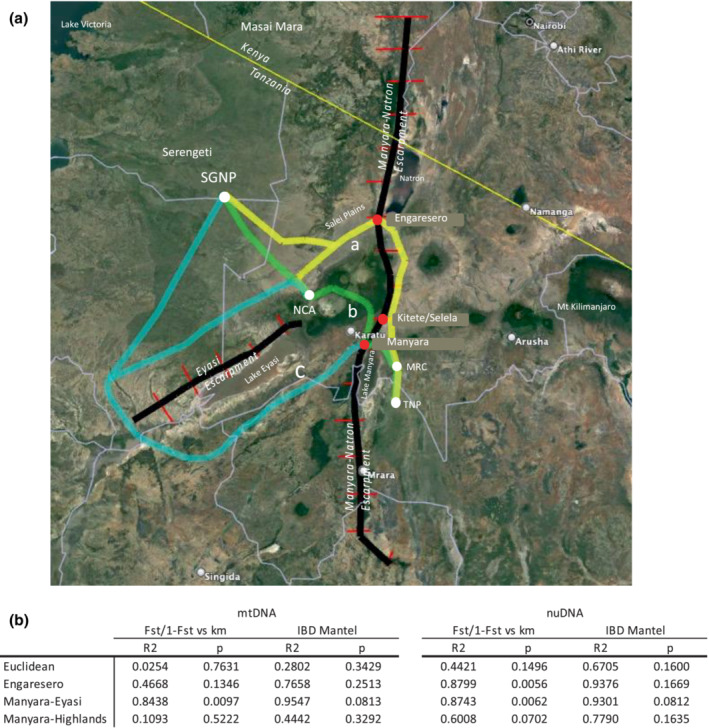
Slope and isolation by distance analysis of the Manyara‐Natron and Eyasi escarpments of the Gregory Rift system. (a). Satellite map (Google Earth) of northern Tanzania and southern Kenya with the Manyara‐Natron and Eyasi escarpments of the Gregory Rift highlighted by black lines. Combined with Ngorongoro highlands, these escarpments pose a formidable barrier to giraffe dispersal between the Serengeti and Tarangire ecosystems. Red lines intersecting the escarpments at 25 km intervals mark the position where maximum slopes were estimated. Maximum slopes were also estimated at 5 km intervals (Table S5 and Figure S5). Yellow lines‐**a** mark the Engaresero‐Salei plains potential dispersal route utilizing the Engaresero WEP (red dot) across the Manyara‐Natron escarpment near Lake Natron and the Salei Plains (2°38′0.87″S 35°52′46.71″E). Green lines‐**b** mark the Manyara‐Highland potential dispersal route utilizing the Manyara WEP (red dot) (3°26′39.87″S 35°48′41.68″E) across the Manyara‐Natron escarpment on near the western shore of Lake Manyara. Blue lines‐**c** mark the Manyara‐Eyasi potential dispersal route utilizing the Manyara WEP (red dot) across the Manyara‐Natron escarpment on near the western shore of Lake Manyara. See Table S5 for maximum slope estimates each of the three dispersal routes. (b) Isolation by distance correlation of pairwise population *F*_*ST*_ for mtDNA and nuDNA between geographic distance (km) of four alternative dispersal routes connecting the Serengeti and Tarangire ecosystems. *R* ^2^ and *p* values shown for Rousset method (*F*_*ST*_/1−*F*_*ST*_ vs. km) and IBD Mantel test. *n* = 6 pairwise comparisons of four populations. **Statistically significant, *p* < .01.

Maximum slopes across the Manyara‐Natron Escarpment averaged 54.5% and ranged between 31.7% and 79.3% (Table S5, Figure S2) whereas the maximum slopes for the Eyasi Escarpment averaged 60.3% for the first 70 km before declining to <6% at its southwest terminus (Table S5). A possible dispersal location across the Manyara‐Natron escarpment, which we denoted as the Engaresero wildlife escarpment pass (WEP) (Figure 7, Figure S3), was identified near the village of Engaresero and Lake Natron (37′02.05″S 35 51′43.45″E), which is located approximately 120 km north of our EMG populations and 70–150 km east of our WMG populations. The Manyara‐Natron escarpment at this point exhibits several interwoven animal tracks across the escarpment leading directly to the Salei Plains with unimpeded access to the Serengeti Ecosystem (Figure 7). We found that the maximum slope of the potential Engaresero WEP traced along the animal tracks across the escarpment was 30.2% (Table S5), which is the lowest maximal slope that we detected along 400 km of the Manyara‐Natron Escarpment. By comparison the maximal slope of the Kitete‐Selela wildlife corridor located near MRC and LMNP was found to be 50.1% (Figure S4, Table S5). The only other region identified with a maximum slope <35% corresponded to a location immediately west of Lake Manyara previously identified as an elephant dispersal route (Douglas‐Hamilton, 1973; Prins et al., 1994) that we denoted as the Manyara WEP (Figure 7a, Figure S5). The maximal slope of the Manyara WEP is 31.6%. Among these three corridors and passes, we speculated that the maximal slopes of the Manyara and Engaresero WEPs are low enough to potentially support giraffe movement whereas the Kitete‐Selela corridor is too steep. Utilizing the Manyara and Engaresero WEP, we mapped three alternative dispersal routes connecting the SGNP and NCA Masai giraffe populations in the west with the MRC, LMNP, and TNP populations in the east (Figure 7a). We denoted these potential giraffe dispersal routes as Engaresero‐Salei, Manyara‐Eyasi, and Manyara‐Highland (Figure 7a).

### 3.5 Landscape genetic connectivity

To evaluate alternative dispersal routes between the four major populations studied (TNP, MRC, NCA, and SGNP), we estimated the correlation between pairwise population *F*_*ST*_ estimates (Table 1, Figure S6) of mtDNA and nuDNA genetic variation with pairwise geographic distances (*n* = 6 pairwise comparisons). We evaluated three potential dispersal routes (Figure 7b) as well as the Euclidean distances between populations using two methods to estimate the relationship of *F*_*ST*_ and geographic distance: the Rousset method—regression of *F*_*ST*_/(1−*F*_*ST*_) on geographic distance (Rousset, 1997a), and the isolation by distance (IBD) Mantel test (Bohonak, 2002). A highly significant correlation between mtDNA *F*_*ST*_ and geographic distance was found for the Manyara‐Eyasi WEP (*R* ^2^ = 0.844**), while significant correlations for nuDNA *F*_*ST*_ were found for the Engaresero‐Salei WEP (*R* ^2^ = 0.880**) and the Manyara‐Eyasi WEP (*R* ^2^ = 0.874**) using the Rousset method (Figure 7b, Figure S6). No significant correlations were seen using the IBD Mantel test, but the relative correlation values of the four alternative dispersal routes were similar to the values from the Rousset method.

## 4 DISCUSSION

The Masai giraffe population has plummeted in the past 30 years as the result of human activities including illegal hunting and land use changes creating fragmented populations with reduced opportunities for dispersal among them (Bolger et al., 2019). In addition to human activities, geographic barriers such as mountains and steep escarpments impede animal movements (Taylor et al., 1972; Wall et al., 2006) and are likely to further constrain dispersal across the Gregory Rift escarpments (GRE). Based on the whole genome mtDNA sequence data, a proxy for female‐mediated gene flow, we found that female‐mediated gene flow of Masai giraffes has likely not occurred across the GRE in the past ~250,000–300,000 years. We base this claim upon (1) the absence of shared mtDNA haplotypes between the major Masai giraffe populations of the Serengeti Ecosystem west of the GRE and the Tarangire Ecosystem east of the GRE, (2) the estimated time (289 kya) of divergence of the western and eastern Masai giraffe (WMG and EMG) haplogroup clades, and (3) the observation that the WMG and EMG haplotypes are no more closely related to each other than they are to South African giraffe mtDNA haplotypes. Our analysis of mtDNA haplotypes of Masai giraffes from three other studies (Agaba et al., 2016; Brown et al., 2007; Coimbra et al., 2021) from Kenya, Zambia, and southern Tanzania confirm the radical separation of mtDNA haplotypes east and west of the GRE. Masai giraffes and South African giraffes arose from a common ancestor of a southern African clade approximately 230 kya (Coimbra et al., 2021), based on phylogenomic analysis of nuclear DNA SNPs among representative Cetartiodactyla species including the giraffe subspecies and okapi. Using a small fragment of the mtDNA from Brown and colleagues the time of divergence of Masai and South African giraffe dates to between 130–370 kya (Brown et al., 2007), bracketing the more recent estimate of Coimbra and colleagues (Coimbra et al., 2021). Whether the EMG, WMG, and South African haplogroups diverged before or after speciation of Masai‐South African giraffes cannot be determined from either our analysis or from recent phylogenetic studies by others (Coimbra et al., 2021; Fennessy et al., 2016; Petzold & Hassanin, 2020) because of highly variable estimates of divergence times. We estimated the EMG‐WMG mtDNA haplotype divergence time to be approximately 289 kya, which would place the EMG‐WMG mtDNA haplotype divergence before the Masai‐South African giraffe speciation estimated to be 230 kya. However, the uncertainty of the time estimates of the subspeciation event and the divergence of the EMG‐WMG mtDNA haplotypes is too large to preclude a definitive order.

The existence of ancient mtDNA haplotype clades within a species is not uncommon. For example, a major mtDNA haplotype of the African savannah elephant is shared with the forest elephant (Ishida et al., 2011, 2013) and is more divergent than the Masai giraffe WMG and EMG haplotypes. However, the forest elephant mtDNA haplotype is present in populations east and west of the GRE (Ahlering et al., 2012; Lohay et al., 2020). That the forest elephant mtDNA haplotype is present in major populations east of the GRE whereas the WMG Masai giraffe haplotype is not, is likely due to the different mobility of these two animals in traversing mountainous terrain. The combination of the giraffe's high anterior center of gravity and elevated forelegs and neck (Mitchell, 2021) makes climbing difficult as can be seen in video recordings of giraffes attempting to climb modest inclines (Nat Geo Wild, 2016). Savannah elephants reportedly still traverse the Manyara‐Natron escarpment through the Kitete‐Selela corridor (Chlebek & Stalter, 2015) and historically crossed this escarpment immediately west of Lake Manyara (Douglas‐Hamilton, 1973; Prins & de Jong, 2022) However, giraffes probably do not use these corridors (Jones et al., 2009). Our assessment of the entire 400 km length of the Manyara‐Natron escarpment found only two locations with maximal slopes below 35%, but giraffes have not been reported to cross over the escarpment at either of these locations. Nonetheless, the nuDNA analysis strongly indicates that gene flow has occurred across the GRE as recent as a few thousand years ago. The absence of mtDNA gene flow across the GRE indicates that females have not been responsible for this genetic exchange.

A separate but related question is the origin of the geographic segregation of WMG and EMG mtDNA haplotypes. We consider two alternative hypotheses: (1) the founders of the western and eastern sides of the GRE were independent, arising from ancestral Masai giraffe populations that already possessed different mtDNA haplotypes and (2) the western side of the GRE was founded first and subsequently the eastern side was founded by dispersal from the west. The independent origin hypothesis would require circumnavigating the steep GRE either from the north between the gap separating the western and eastern branches of the Great Rift in Uganda and Kenya or possibly from the south through gaps in the Tanzanian Craton in southern Tanzania and Zambia (le Gall et al., 2004; Saria et al., 2014; Scoon, 2018). After the independent colonization of the eastern and western populations, occasional male dispersal over the GRE would be sufficient to explain the current level of nuDNA genetic differentiation between the Serengeti and Tarangire ecosystems. Although we favor the independent origin hypothesis, we have insufficient data to critically evaluate these two alternative hypothesis, but population genetic analysis of Masai giraffe populations in southern Tanzania and southern Kenya may provide clues to the origin of western and eastern Masai giraffes.

Population genetic analysis of nuclear DNA also shows strong genetic differentiation of western and eastern Masai giraffes. Structure analysis indicates very little admixture between EMG and WMG populations but considerable admixture between SGNP and NCA populations in the west and between TNP and MRC in the east. Similarly, PCA analysis shows that the first principal component (PC1) is positive for all eastern giraffes and negative for all western giraffes. While the *F*_*ST*_ values between western SGNP and NCA and between eastern MRC and TNP are correlated with distance between populations, the highest pairwise *F*_*ST*_ among all four populations is seen between MRC and NCA, which paradoxically are the closest cross‐GRE populations. However, the imposing Ngorongoro Highlands and Manyara‐Natron escarpment would likely preclude giraffe movements directly between the MRC and NCA populations. We identified two alternative routes, denoted as Manyara‐Eyasi and Engaresero, that circumvent the steepest escarpment slopes and for which the steepest slopes do not exceed 32%. These alternative routes entail a substantial increase in distance connecting eastern and western Masai giraffe populations compared to Euclidean distance or a route directly over the Ngorongoro Highlands. We reasoned that dispersal routes that exhibited the highest degree of correlation between *F*_*ST*_ and geographic distance would be the most likely dispersal routes that have been used in the past, consistent with the theory of isolation by distance originally proposed by (Wright, 1943) and further elaborated by others (Bohonak, 2002; Rousset, 1997a; Slatkin, 1993). The Manyara‐Eyasi and Engaresero dispersal routes showed highly significant correlation between nuDNA *F*_*ST*_ and geographic distance whereas the Euclidean distance and Manyara‐Highlands routes did not. We postulate that the Manyara‐Eyasi and Engaresero routes may have served as giraffe dispersal routes in the distant past. However, giraffes are unlikely to have used these routes in recent decades due to anthropogenic changes in the areas above and below the Manyara‐Natron escarpments at these locations (Bond et al., 2017; Caro & Davenport, 2016; Jones et al., 2009; Lamprey, 1964; Lee & Bolger, 2017; Prins & de Jong, 2022).

The pairwise nuDNA *F*_*ST*_ values between eastern and western Masai giraffe populations range between 0.0773 and 0.0832, which are comparable to the difference between plains zebra populations in Etosha NP and Luangwa Valley NP separated by 1800 km (Larison et al., 2021) and larger than gray wolf (*Canis lupus*) populations in British Columbia and Alaska separated by 2000 km of mountainous terrain and between wolf populations in Russia separated by 2100 km (Pacheco et al., 2022). The time of separation for wolf populations can be calibrated with the estimated time of the last glacial maximum (34.4 kya) and the flooding of the Bering Land Bridge (ca. 11 kya), which separated gray wolf populations in Russia and North America (Pacheco et al., 2022). There are no similar geological events that are known to have significantly altered the escarpments of the Gregory Rift that we can use to estimate the time of divergence between western and eastern Masai giraffes. If we assume that the mutation‐substitution rate of giraffes and wolves is approximately the same as they are for other mammals, then we can derive an estimated time of separation of WMG and EMG at approximately 10 kya. This estimate could be off by a factor of two or more due to differences in the mating system and dispersal characteristics of giraffe, which may be quite different from plains zebra and gray wolf. However, it does suggest that WMG and EMG have been reproductively isolated by at least a thousand years and well before the Anthropocene. Coupled with the mtDNA analysis, which suggests that female‐mediated gene flow has not occurred between WMG and EMG <~289 kya, we conclude that western and eastern Masai giraffe are reproductively isolated.

The patterns of population genetic differentiation of nuDNA within the Serengeti and Tarangire ecosystems also revealed reduced genetic connectivity. Because the mutation‐substitution rate of mtDNA is much higher than nuDNA (Allio et al., 2017), *F*_*ST*_ values for mtDNA are generally much larger than for nuDNA and this was found to be true for population pairwise *F*_*ST*_ values in this study with the exception of MRC‐TNP where nuDNA *F*_*ST*_ was ~6‐fold higher than mtDNA *F*_*ST*_. This finding is consistent with the hypothesis of recent loss of genetic connectivity, because extant nuDNA variation will be more rapidly impacted by random genetic drift than extant mtDNA variation.

Masai giraffes were suggested to exhibit a relatively high level of inbreeding compared with other giraffe species based on the analysis of one individual from the Maasai Mara in Kenya and five individuals from the Selous Game Reserve in southern Tanzania (Coimbra et al., 2021). We found that individual inbreeding coefficients (*F*) for Masai giraffe in our study were high, comparable to the *F* of cheetah (*Acinonyx jubatus jubatus*) (Prost et al., 2022) located in South Africa (*F* = 0.104) utilizing the same method for estimating *F* (Vieira et al., 2013). Moreover, the average inbreeding coefficient for the western Masai giraffe populations was substantially larger than the eastern Masai giraffe populations. Correlated with our finding of high inbreeding coefficients for WMG, *F*_*ST*_ values between the Serengeti and NCA populations in this study and among Serengeti populations studied by Brown and coworkers are relatively high (Brown et al., 2007). The high level of *F* seen in the Masai giraffe is likely associated with severe population decline caused by the recurrent rinderpest epidemics that swept across the African continent from 1890s to the 1960s that resulted in massive mortality of cattle, wildebeests, Cape buffalos (*Syncerus caffer*), common elands (*Taurotragus oryx*), and giraffes (Plowright, 1982). The relatively high inbreeding values in Masai giraffes may have resulted from extreme population bottlenecks, population fragmentation, and random drift caused by the rinderpest epidemic. Rinderpest has impacted giraffe populations directly through infection (Plowright, 1982) and indirectly through fire as a result of lower grazing pressure, increasing the fuel load and the subsequent loss of woody browse (Sinclair, 2012). Although rinderpest infections were prevalent east and west of the GRE, the higher wildebeest density in the Serengeti Ecosystem and the greater buffalo density in the Tarangire Ecosystem (Lamprey, 1964) may indicate differential direct and indirect effects of rinderpest on the western and eastern Masai giraffe populations. Giraffe numbers in the Serengeti and Tarangire Ecosystems did not rebound until the 1970s after widespread cattle vaccinations suppressed the spread of rinderpest to wildlife (Plowright, 1982; Sinclair, 2012). Unfortunately, giraffe census data were not collected before and after the rinderpest epidemic to determine if these two ecosystems were impacted to a different degree, and we have no direct evidence that rinderpest affected the inbreeding levels and population differentiation of Masai giraffes. Alternatively, the mating system and reproductive behavior of giraffes may have resulted in high inbreeding and population differentiation. Currently, there is little information about giraffe mating behavior to determine how philopatry vs. dispersal might influence population genetic structure and relatedness (Bercovitch & Deacon, 2015; Bond, Lee, et al., 2021).

### 4.1 Masai giraffe conservation

We have shown compelling evidence that eastern and western Masai giraffes are reproductively isolated and have been so for thousands of years. The apparent reason for their genetic separation is the formidable Gregory Rift Escarpments with maximal slopes that average 50% across a 400+ km extent and only a few passes with slopes between 31%–40%. Therefore, we propose that the Masai giraffe population estimated to be 35,000 should now be considered as two separate evolutionary significant units (ESU) with no more than 20,000 in each. The proposed designation of western Masai giraffe and eastern Masai giraffe as ESUs is based on meeting the specific criteria that the two populations in question are reproductively isolated (Waples, 1995) and that genetically they are “reciprocally monophyletic for mtDNA alleles and show significant divergence of allele frequencies at nuclear loci” (Moritz, 1994). Considering the western Masai giraffe and eastern Masai giraffe as distinct ESUs has important implications for their conservation.

Masai giraffes appear to have rarely traversed the GRE over their evolutionary history, and it is impractical to develop wildlife corridors across the escarpments that could be used by giraffes to genetically reconnect western and eastern populations. Therefore, conservation efforts should be focused on maintaining and developing corridors among the populations within the eastern Masai giraffe population and within the western Masai giraffe population, as separate but coordinated efforts. The challenges faced by WMG and EMG are also quite different. For WMG, numerous populations are scattered across the ~30,000 km^2^ Serengeti Ecosystem, with no natural barriers that should impede dispersal and gene flow between them, but dispersal and movement rates among WMG populations are unknown. WMG populations are subject to predation pressure and illegal hunting (Rentsch et al., 2015; Strauss et al., 2015) despite the relatively high degree of protection afforded to national parks. By contrast the eastern Masai giraffe that inhabit the Tarangire Ecosystem east of the GRE is highly fragmented by tarmac roads, towns, villages, agriculture, and pastoralism all which have expanded exponentially in the past few decades (Borner, 1985; Lamprey, 1964; Morrison et al., 2016; Mwalyosi, 1991). Based on triannual surveys of individually identified Masai giraffes in the Tarangire Ecosystem, significant dispersal and movement among protected areas in the Tarangire Ecosystem was observed as recently as 2017 (Bond, König, et al., 2021; Lavista Ferres et al., 2021; Lee & Bolger, 2017). The two largest giraffe populations in the Tarangire Ecosystem are located in the TNP and MRC (Lee & Bolger, 2017) whose boundaries are only 4 km apart. In the past the TNP‐MRC‐Lake Natron wildlife corridor provided connectivity between MRC and TNP, but this corridor has seen a recent dramatic increase in agriculture and human populations potentially reducing wildlife dispersal (Jones et al., 2009; Kikoti, 2009; Lohay et al., 2022; Msoffe et al., 2011). This corridor is bisected by a major tarmac highway (A104) that wildlife must cross to move between TNP and MRC and much of the expansion of human activities in the Tarangire Ecosystem has occurred along this road. Maintaining the TNP‐MRC‐Lake Natron corridor to maintain genetic connectivity between EMG populations in the TNP and MRC is extremely important. We recommend that agriculture and contiguous human settlements be restricted in the areas between these populations, and that speed bumps be installed on the A104 highway and wildlife bridges across it be considered.

The small Masai giraffe population in Lake Manyara National Park (ca.100 animals) (Lavista Ferres et al., 2021) is particularly vulnerable because of the small size of the dry land available to herbivores (<100 km^2^). LMNP is comprised of a narrow strip of land sandwiched between the Manyara‐Natron Escarpment to the west and Lake Manyara to the east. Wildlife dispersal routes around the southwest and northeast ends of Lake Manyara linking to other areas of the Tarangire Ecosystem (Lamprey, 1964) are now largely blocked by agriculture and townships including the rapidly growing town of Mto Wa Mbu. Movement and social network studies have found that the Masai giraffe population in LMNP is highly isolated, despite the proximity to substantial populations in the MRC and TNP (Lavista Ferres et al., 2021; Lee & Bolger, 2017). Genetically, LMNP giraffes are unique in displaying WMG and EMG mtDNA haplotypes and intermediate nuDNA frequencies between the eastern and western Masai giraffes. The absence of WMG mtDNA haplotypes in nearby TNP and MRC suggests that female‐mediated gene flow from LMNP to TNP and MRC does not occur. Conversely, the much closer correlation of nuDNA allele frequencies as shown by admixture and principal component analysis of LMNP and EMG suggest that gene flow from TNP and/or MRC to LMNP has occurred in the past. This asymmetry is germane to the two alternative hypotheses for the admixture of eastern and western Masai giraffe mtDNA and nuDNA present in LMNP giraffes: (1) LMNP was originally founded by WMG then subsequently received migrants from EMG or (2) LMNP was originally found by EMG and subsequently received migrants from WMG. Because LMNP giraffes have a higher proportion of EMG nuDNA admixture, the eastern origin appears more likely. However, the study of the evolution of the Lake Manyara Basin has shown that over the past 10,000 years Lake Manyara was much larger and may have extended all the way to Lake Natron (Bachofer et al., 2014, 2018). This would have imposed a dispersal barrier between giraffes in the narrow strip of land between the GRE and Lake Manyara and giraffes in the Tarangire Ecosystem lying east of the lake until relatively recently (i.e., in the past hundreds to thousands of years). Therefore, we believe it is possible that LMNP was originally founded by western Masai giraffes dispersing down the escarpment, and then later experienced substantial nuDNA and mtDNA introgression from eastern Masai giraffes after Lake Manyara receded. Northern and southern access around Lake Manyara, however, has been blocked in the past few decades, terminating gene flow between LMNP and other areas of the Tarangire Ecosystem. The population genetic analysis of the LMNP giraffes suggests that this population is currently healthy, but vulnerable to stochastic events such as the emergence of infectious diseases, a major climatic event influencing resource availability/distribution or a major geological event that alters the landscape.

## AUTHOR CONTRIBUTIONS

**George G. Lohay:** Conceptualization (equal); data curation (lead); formal analysis (equal); investigation (equal); methodology (equal); project administration (supporting); resources (equal); supervision (supporting); visualization (equal); writing – original draft (equal); writing – review and editing (supporting). **Derek E. Lee:** Investigation (supporting); writing – original draft (supporting); writing – review and editing (supporting). **Lan Wu‐Cavener:** Formal analysis (supporting); resources (supporting); writing – review and editing (supporting). **David L. Pearce:** Formal analysis (supporting); methodology (supporting); software (supporting); writing – review and editing (supporting). **Monica L. Bond:** Writing – original draft (supporting); writing – review and editing (supporting). **Xiaoyi Hou:** Resources (supporting); writing – review and editing (supporting). **Douglas R. Cavener:** Conceptualization (lead); data curation (equal); formal analysis (equal); funding acquisition (lead); investigation (equal); methodology (equal); project administration (lead); resources (equal); supervision (lead); validation (lead); visualization (equal); writing – original draft (equal); writing – review and editing (equal).

## BENEFIT‐SHARING STATEMENT

Benefits Generated: A research collaboration was developed with scientists from the Tanzania providing genetic samples, all collaborators are included as co‐authors and/or acknowledged for their contributions, and the research addresses a priority concern, in this case the conservation of Masai giraffe in Tanzania and more broadly giraffe East Africa. Our group is committed to international scientific partnerships, as well as institutional capacity building.

## RESEARCH PERMITS

Permission to conduct this research was provided by the Commission of Science and Technology #2020‐185‐NA‐1990‐172, Tanzania Wildlife Research Institute, Tanzania National Park Authority, Ngorongoro Conservation Authority, Manyara Ranch Conservancy, and IACUC# PROTO201901219 by Penn State University.

## Supporting information


Appendix S1.

## Data Availability

Mitochondrial DNA sequences for 1140 bp can be accessed using GenBank Accession Number: OP442601–OP442932. Whole genome sequence data for the mitochondrial has been submitted to GenBank (GenBank Submissions grp 8715258). Dryad, 10.5061/dryad.m905qfv4h.
