# Supplementary material for: Genetic evidence of population subdivision among Masai giraffes separated by the Gregory Rift Valley in Tanzania
Source: Ecol Evol. 2023 Jun 12;13(6):e10160. doi: 10.1002/ece3.10160 (PMC10259769; doi:10.1002/ece3.10160)
Supplement: Supplementary file 1 — Appendix S1. [file ECE3-13-e10160-s001.docx]

*Supporting Information for Online Publication*

Table S1.  Genetic parameters estimated at each locality for mitochondrial DNA (1140bp) for the Masai giraffes.

| **Location** | **Pop code** | **GPS coordinates** | **N** | **N_H_** | **H_d_** | **π(%)** |
| --- | --- | --- | --- | --- | --- | --- |
| **Burunge Wildlife Management Area** | BWMA | 3.959° S, 35.809° E | 21 | 2 | 0.638 | 0.066 |
| **Lake Manyara National Park** | LMNP | 3.4459° S, 35.8093° E | 23 | 4 | 0.654 | 0.292 |
| **Tarangire National Park** | TNP | 4.0057° S, 35.9788° E | 51 | 5 | 0.495 | 0.066 |
| **Manyara Ranch Conservancy** | MRC | 3.5846° S, 36.0021° E | 96 | 3 | 0.602 | 0.061 |
| **Ngorongoro Conservation Area** | NCA | 3.2279° S, 35.5075° E | 74 | 4 | 0.464 | 0.157 |
| **Serengeti National Park** | SGNP | 2.3333° S, 34.8333° E | 55 | 7 | 0.537 | 0.107 |

N= number of sequences from fecal and tissue samples, N_H_=Number of haplotypes, H_d_=Haplotype diversity and π= haplotype diversity in %.

Table S2. Mitochondrial DNA haplotypes for Masai Giraffes recorded in this study. Haplotype names contains information of where the haplotype was found whether from West or East Masai Giraffe population. Nine haplotypes have been published (Brown, Brenneman, Koepfli, Pollinger, Mila, et al., 2007; Coimbra et al., 2021) and 13 new haplotypes found from this study.

| **Haplotypes** | **BWMA** | **TNP** | **MRC** | **LMNP** | **NCA** | **SGNP** | **Total** | **GenBank Accession No.** |
| --- | --- | --- | --- | --- | --- | --- | --- | --- |
| EMG1 | 11 | 35 | 51 | 2 |  |  | 99 | OP442601 |
| EMG9 | 6 | 10 | 30 | 5 |  |  | 51 | OP442602 |
| EMG4 | 4 | 2 | 15 |  |  |  | 21 | OP442603 |
| WMG1 |  |  |  | 12 | 53 | 36 | 101 | OP442604 |
| WMG3 |  |  |  |  | 18 | 15 | 33 | OP442605 |
| EMG7 |  | 3 |  |  |  |  | 3 | OP442606 |
| EMG8 |  | 1 |  |  |  |  | 1 | OP442607 |
| WMG4 |  |  |  |  |  | 2 | 2 | OP442608 |
| WMG5 |  |  |  |  |  | 1 | 1 | OP442609 |
| WMG2 |  |  |  |  | 2 |  | 2 | OP442610 |
| WMG12 |  |  |  |  |  | 1 | 1 | OP442611 |
| WMG13 |  |  |  |  |  | 1 | 1 | OP442612 |
| EMG16 |  |  |  | 4 |  |  |  | OP442613 |
| Total | 21 | 51 | 96 | 23 | 73 | 56 | 320 |  |

Table S3. Mitochondrial whole genome pairwise genetic distances for representatives of each of the Southern Giraffe subspecies group.

Table S4. Mitochondrial (mtDNA) haplotype frequencies based on a 652-bp sequence fragment common to this study and to other studies. Athi, Chyulu and Naivaisha are from south-central Kenya (Brown et al. 2007), Selous Game Reserve (SGR) are from southeast Tanzania and Thorncroft’s giraffe samples are from the Luangwa Valley National Park (LVNP) in Zambia (Coimbra et al. 2021)

| **Haplotype ID** | **ATHI** | **BWMA** | **CHYULU** | **LMNP** | **LVNP** | **MRC** | **NCA** | **NAIVASHA** | **SGNP** | **TNP** | **SGR** | **Total** |
| --- | --- | --- | --- | --- | --- | --- | --- | --- | --- | --- | --- | --- |
| EMG1.C |  | 1 |  |  |  | 1 |  |  |  | 8 |  | 10 |
| EMG1.A |  |  |  |  |  | 1 |  |  |  |  |  | 1 |
| EMG2.B |  | 2 |  |  |  |  |  |  |  |  |  | 2 |
| EMG2.D |  |  |  |  |  |  |  |  |  | 1 |  | 1 |
| EMG4 |  |  | 2 | 2 |  | 2 |  | 2 |  | 2 |  | 10 |
| EMG5 |  | 2 |  |  |  |  |  |  |  | 10 |  | 12 |
| EMG9.G | 5 | 5 | 12 | 6 | 0 | 24 | 0 | 4 | 0 | 17 | 5 | 78 |
| WMG1.C |  |  |  | 7 |  |  | 9 |  | 17 |  |  | 33 |
| WMG1.M |  |  |  |  |  |  | 2 |  | 2 |  |  | 4 |
| WMG2 |  |  |  |  |  |  |  |  | 2 |  |  | 2 |
| WMG3.C |  |  |  |  |  |  |  |  | 4 |  |  | 4 |
| WMG3.B |  |  |  |  |  |  |  |  | 2 |  |  | 2 |
| EMG8 |  |  |  |  |  |  |  |  |  | 3 |  | 3 |
| WMG5.B |  |  |  |  |  |  |  |  | 1 |  |  | 1 |
| WMG5.A |  |  |  |  |  |  |  |  | 1 |  |  | 1 |
| WMG4.A |  |  |  |  |  |  | 1 |  |  |  |  | 1 |
| WMG4.B |  |  |  |  |  |  | 8 |  |  |  |  | 8 |
| LVNP8-14 |  |  |  |  | 6 |  |  |  |  |  |  | 6 |
| AthiM18 | 2 |  |  |  |  |  |  |  |  |  |  | 2 |
| AthiM8 | 10 |  |  |  |  |  |  |  |  |  |  | 10 |
| AthiM25 | 1 |  |  |  |  |  |  |  |  |  |  | 1 |
| ChyuluM25 |  |  | 1 |  |  |  |  |  |  |  |  | 1 |
| Lobo2064 |  |  |  |  |  |  |  |  | 1 |  |  | 1 |

Table S5. Geographic slope analysis of known and potential wildlife dispersal routes across the Manyara-Natron and Eyasi escarpments of the Gregory Rift system. GPS coordinates mark the eastern side of the escarpment. The 250km transect is 1.8km from Manyara WEP. The 350km transect near the end of the Manyara-Natron escarpment may allow giraffe movement but giraffe would still need to circumnavigate the Lake Eyasi and the Eyasi escarpment to reach the Serengeti ecosystems in the west.

Table S6. Individual inbreeding coefficients, *F*, for each of the 100 individual Masai giraffe. Inbreeding coefficients were estimated using the program NgsF (Vieira *et al.*, 2013) .

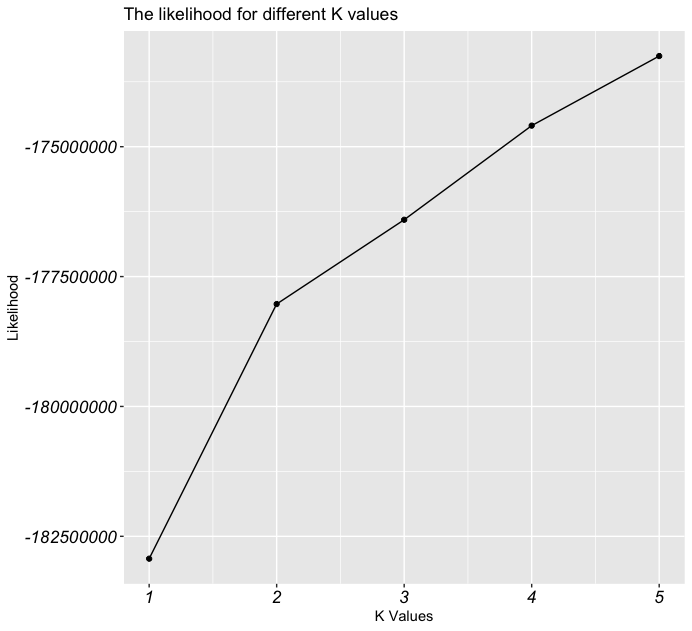


Fig. S1. Plot to determine K for admixture analysis. K=2 occurs at the point where the largest change in slope is seen.

Fig.S2 Maximal percent slope estimates along the Manyara-Natron escarpments of the Gregory Rift system. Maximal slopes were estimated from elevation profiles across the escarpment at 5km intervals beginning in Kenya at 0 Km (1°15'4.45"S 36° 2'46.30"E) and terminating in Tanzania at 400km (4°43'46.27"S 35°57'11.86"E). Data from 305-400km are not shown in this figure.

Fig. S3. Engaresero-Salei potential wildlife dispersal route over Manyara-Natron escarpment of the Gregory Rift near the town of Engaresero and Lake Natron. The blue line shows the least resistance path.

Fig.S4 Kitete-Selela wildlife corridor. White lines indicate boundaries of the corridor. Blue line is the approximate location of minimal slope track across the escarpment. The blue line shows the least resistance path.

Fig. S5. Potential route over the Manyara-Natron escarpment near the western shore of Lake Manyara and Endallah immediately above the escarpment. This area has been a major wildlife dispersal route over the escarpment prior to the extensive development of agriculuture over the past fifty years. The minimal slope path (blue line) is the eastern terminus of the proposed Manyara-Eyasi giraffe dispersal route connecting Western Masai giraffe and Eastern Masai giraffe.

Fig. S6. Population pairwise genetic and geographic distances (a) for four populations MRC, TNP, NCA, and SGNP and four possible dispersal routes as shown in Fig. 7a. Regression analysis of four representative dispersal routes are shown in b-e. Statistical results for all routes for mtDNA and nuDNA *F_ST_* /1- *F_ST_* are shown in Fig. 7b. Blue dots = pairwise data point; red dots = predicted Y values from the regression.
